# SerpinB3 as a Pro-Inflammatory Mediator in the Progression of Experimental Non-Alcoholic Fatty Liver Disease

**DOI:** 10.3389/fimmu.2022.910526

**Published:** 2022-07-08

**Authors:** Erica Novo, Andrea Cappon, Gianmarco Villano, Santina Quarta, Stefania Cannito, Claudia Bocca, Cristian Turato, Maria Guido, Marina Maggiora, Francesca Protopapa, Salvatore Sutti, Alessia Provera, Mariagrazia Ruvoletto, Alessandra Biasiolo, Beatrice Foglia, Emanuele Albano, Patrizia Pontisso, Maurizio Parola

**Affiliations:** ^1^Department of Clinical and Biological Sciences, Unit of Experimental Medicine and Clinical Pathology, University of Torino, Torino, Italy; ^2^Department of Medicine, University of Padova, Padova, Italy; ^3^Department of Surgical, Oncological and Gastroenterological Sciences – DISCOG, University of Padova, Padova, Italy; ^4^Department of Molecular Medicine, University of Pavia, Pavia, Italy; ^5^Department of Health Sciences, University of Piemonte Orientale, Novara, Italy

**Keywords:** NASH, SerpinB3, macrophages, innate immunity, hepatocytes

## Abstract

Non-alcoholic fatty liver disease (NAFLD) is becoming the most common chronic liver disease worldwide. In 20-30% of patients, NAFLD can progress into non-alcoholic steatohepatitis (NASH), eventually leading to fibrosis, cirrhosis and hepatocellular carcinoma development. SerpinB3 (SB3), a hypoxia-inducible factor-2α dependent cysteine protease inhibitor, is up-regulated in hepatocytes during progressive NAFLD and proposed to contribute to disease progression. In this study we investigated the pro-inflammatory role of SB3 by employing phorbol-myristate acetate-differentiated human THP-1 macrophages exposed *in vitro* to human recombinant SB3 (hrSB3) along with mice overexpressing SB3 in hepatocytes (TG/SB3) or knockout for SB3 (KO/SB3) in which NASH was induced by feeding methionine/choline deficient (MCD) or a choline-deficient, L-amino acid defined (CDAA) diets. *In vivo* experiments showed that the induction of NASH in TG/SB3 mice was characterized by an impressive increase of liver infiltrating macrophages that formed crown-like aggregates and by an up-regulation of hepatic transcript levels of pro-inflammatory cytokines. All these parameters and the extent of liver damage were significantly blunted in KO/SB3 mice. *In vitro* experiments confirmed that hrSB3 stimulated macrophage production of M1-cytokines such as TNFα and IL-1β and reactive oxygen species along with that of TGFβ and VEGF through the activation of the NF-kB transcription factor. The opposite changes in liver macrophage activation observed in TG/SB3 or KO/SB3 mice with NASH were associated with a parallel modulation in the expression of triggering receptor expressed on myeloid cells-2 (TREM2), CD9 and galectin-3 markers, recently detected in NASH-associated macrophages. From these results we propose that SB3, produced by activated/injured hepatocytes, may operate as a pro-inflammatory mediator in NASH contributing to the disease progression.

## Introduction

Non-Alcoholic Fatty Liver Disease (NAFLD) is rapidly becoming the major cause of chronic liver disease (CLD) worldwide, with epidemiological data indicating a 25% prevalence in the general population and an even higher prevalence in obese and Type II diabetes patients (1–5). The term NAFLD encompasses a spectrum of liver diseases ranging from simple hepatic steatosis or NAFL, without significant evidence of hepatocellular damage, to non-alcoholic steatohepatitis (NASH) characterized by cell damage (ballooning), cell death and lobular inflammation, with or without fibrosis. NASH can develop in at least 20-30% of patients and can progress to liver cirrhosis and associated complications (1–5), including the development of hepatocellular carcinoma (HCC) which, in turn, can also develop in non-cirrhotic patients (6–8). At present the mechanisms underlying the progression of NAFLD towards more advanced stages of the disease are only partially defined and validated therapies for this liver disease are not currently available, although several drugs are continuously evaluated in clinical trials and novel therapeutic approaches are being designed (9–14).

A major issue favoring NAFLD progression is represented by the chronic activation of inflammatory response, which involves either macrophages originating from resident hepatic Kupffer cells or CCR2^+^/Ly-6C^hi^ monocyte-derived macrophages (MoMFs) recruited from peripheral blood (15–17). In particular, during NAFLD progression MoMFs acquire peculiar pro-inflammatory, pro-angiogenic and pro-fibrogenic phenotype which plays a major role in sustaining the activation of hepatic stellate cells (HSCs) and other precursor cells into hepatic myofibroblasts (MFs) by the release of several mediators (15–17). More recently, different laboratories have described the emergence, during NAFLD progression and/or in condition of altered lipid metabolism, of a peculiar macrophage phenotype characterized by the expression of Triggering Receptor Expressed on Myeloid cells-2 or TREM2, CD9 and CD68 (18–21), sometimes referred to as NASH-associated macrophages (NAMs) or hepatic lipid-associated macrophages (hepatic LAMs).

The critical persistent activation of hepatic macrophages in NAFLD progression is believed to represent the consequence of complex interactions between growth factors, cytokines, chemokines, reactive oxygen species (ROS) and other less characterized mediators (13–16). Of interest, it has been proposed that NAFLD progression may be closely related to the development of hepatic hypoxia, a tissue event believed to sustain angiogenesis, fibrogenesis as well as inflammatory response through hypoxia-inducible factors (HIFs) - dependent release of specific mediators (16, 22, 23). Along these lines, recent data have outlined the hepatocytes production of histidine-rich glycoprotein (HRG) and SB3 in response to HIF2α-dependent signals (16, 23–26). SerpinB3 is a protein belonging to the family of serine-protease inhibitors (27) which is specifically expressed by hepatocytes and although virtually undetectable in healthy human and murine livers it is greatly up-regulated in liver biopsies from patients with CLD (28–31). Recently, we provided evidence that SB3 can exert a pro-fibrogenic role in the progression of experimental CLD, including experimental NAFLD, by directly acting on activated, myofibroblast-like, hepatic stellate cells (HSC/MFs) in which leads to the up-regulation of major genes involved in fibrogenesis (18). In the present study we take advantage of mice genetically manipulated to carry hepatocyte-specific SB3 overexpression or deletion to provide mechanistic evidence that this protease inhibitor can contribute to NAFLD progression also by acting as a pro-inflammatory mediator.

## Materials and methods

### Materials

The methionine and choline-deficient (MCD) diet, the choline-deficient aminoacid-refined (CDAA) diet, as well as the related control diets, methionine choline supplemented (MCS) diet and choline-supplemented and aminoacid-refined (CSAA) diet were provided by Laboratorio Dottori Piccioni srl (Gessate, Milano, Italy). Enhanced chemiluminescence (ECL) reagents, nitrocellulose membranes (Hybond-C extra) were from Amersham Pharmacia Biotech Inc. (Piscataway, NJ, USA). The following reagents were purchased from Merck Life Science (Milan, Italy): sodium chloride (NaCl), bovine serum albumin (BSA), ethanol, phosphate saline buffer (PBS), citric acid, sodium citrate, hydrogen peroxide, TWEEN, Mayer’s hematoxylin, sodium azide (NaN3), TRI reagent, diaminobenzidine (DAB), Entellan^®^ Slide Mounting Medium, inhibitor BAY-117082 and primers used in Real-Time PCR reactions. The kit for RNA retro-trascription and for real time PCR were purchased from BioRad (Berkeley, CA, USA). Quantitative RT-PCR analyses were carried out using the MiniOpticon™ Real-Time PCR Detection System instrument of the BioRad company (BioRad, Milan, Italy), which also supplied the EvaGreen master mix reagent. Human recombinant SerpinB3 (hrSB3) was produced in our laboratory, as previously described (28). The antibodies used in immunohistochemistry for F4/80 and for Galectin-3 were purchased from Thermofisher Scientific (Waltham, MA, USA) and R&D system (Minneapolis, MN, USA), respectively. Antibody for western blot analysis for anti NFkB 1:1000 (cod. sc-372), anti IL1β 1:1000 (cod. sc-7884) were raised in rabbit and purchased from Santa Cruz Biotechnology (Santa Cruz, CA, USA), whereas anti p-IkB 1:1000 (cod. 9246S), anti IkB 1:1000 (cod. 4814S) were raised in mice and purchased from Cell Signaling (Massachusetts, USA). Secondary antibodies, goat anti-rabbit (cod. 1706515) and goat anti-mouse (cod. 1706516), were purchased from BioRad (Berkeley, CA, USA).

### Methods

#### Animal Experimentation

The role of SB3 in NAFLD/NASH progression was evaluated by employing two different types of genetically manipulated mice (n=8 for each experimental group): 1) C57Bl6J mice that overexpress human SB3 (31, 32), thereafter referred to as TG/SB3 mice. Briefly, these mice were generated by Professor G. Cassani (Technogen S.c.p.A, Piana di Monte Verna, CE, Italy) by inserting the sequence of human SerpinB3 (-7/+1238) into the pcDNA3 plasmid vector under the alpha1-antitrypsin promoter. The expression of SerpinB3 mRNA, as assessed in different organs, was shown to be high in brain, lung and liver, while it was low in the genitals and irrelevant in kidney and muscle (33). Moreover, further studies showed that within the liver the protein expression was limited to hepatocytes, whereas in other organs was detectable in cells of monocytic origin (34, 35); 2) BalbC mice deficient of the reactive site loop of Serpinb3a gene (KO/SB3 mice) (36) were a kind gift of Dr. Gary Silverman and Dr. Cliff J. Luke (University of Pittsburg, Children’s Hospital, Pittsburg, PA) and were originated in a Balb/C mice background, as detailed in ref. 36. Wild-type (WT) strain-matched C57Bl6J and BalbC mice, 8 weeks old, were used as controls and were purchased by Charles River Laboratories (Charles River UK Ltd., Margate, UK). To induce NASH mice were fed on two distinct dietary regimens: a) methionine and choline deficient (MCD) diet for 8 weeks or a choline-deficient, L-amino acid defined (CDAA) diet for 12 weeks. The respective reference diets were methionine and choline supplemented (MCS) diet or choline-supplemented, L-amino acid defined (CSAA). All animals were kept in specific, pathogen free conditions and kept with free access to food and water at the authorized animal house of the Interdepartmental Center of Experimental Surgery of the University of Padova.

#### Assessment of Liver Injury

Livers were rapidly removed, rinsed in ice-cold saline and cut in pieces. Aliquots were immediately frozen in liquid nitrogen and kept at −80°C until analysis. Two portions of the left lobe from each liver were fixed in 10% formalin for 24h and embedded in paraffin. Blood was obtained from tail vein and plasma was separated for ALT determination. Liver sections (4-µm thick) were stained with hematoxylin/eosin using a Roche Ventana HE 600 automatic staining system (Roche Diagnostics International AG, Rotkreuz, Switzerland), while collagen deposition was detected by Picro-Sirius Red staining. Liver sections were scored blindly for steatosis and lobular inflammation, as previously described (24).

#### Cell Lines and Culture Conditions

*In vitro* experiments described in the present study were performed on the following three different macrophage cell culture models: 1) undifferentiated peripheral blood human monocytes obtained from healthy donors and isolated by centrifugation on Ficoll-Paque solution, seeded on Percoll 46% vol/vol solution (Amersham Biosciences) in RPMI 1640 medium containing 10% FCS and 4mM HEPES buffer. Briefly, monocytes were harvested, re-suspended in medium containing 2% FCS and separated from contaminating lymphocytes by adherence to plastic (1h at 37°C). Adherent monocytes were then washed with medium to remove residual non-adherent cells. The percentages of CD134^+^ cells were higher than 98%; 2) undifferentiated human monocytes of the THP-1 cell line, acquired from the American Type Culture Collection (ATCC, Manassas, VA 20108 USA); 3) human monocytes of the THP-1 cell line that were differentiated into macrophages by treatment for 48h with phorbol 12-myristate 13-acetate (PMA, 50 nM). THP-1 cells were cultured in RPMI medium containing 10% fetal bovine serum, 100 U/ml of penicillin, 100 μg/ml of streptomycin and 25 μg/ml of Amphotericin-B (Merck Life Science, Milan, Italy). The differentiated THP-1 cells, after 24h of incubation with fresh culture medium, were stimulated with hrSB3 (200 ng/ml); in some experiments the cells were pre-treated with the IKK protein inhibitor BAY-117082 and then treated with hrSB3 (200 ng/ml).

#### Immunohistochemistry, Sirius Red Staining and Histomorphometric Analysis

Immunohistochemistry analyses were performed on murine liver sections obtained by mice fed on MCD or CDAA diets. Immunostaining procedure was as previously described (24). Briefly, paraffin sections (4 μm thick), mounted on poli-L-lysine coated slides, were incubated with the monoclonal antibody against murine F4/80 (cod. 14-4801-82; Ebioscience, CA, USA; dilution 1:500) or with the polyclonal antibody against murine Galectin-3 (goat anti mouse, cod. AF1197, Biotechne/R&D System, MN, USA; dilution 1:50). After blocking endogenous peroxidase activity with 3% hydrogen peroxide and performing microwave antigen retrieval in sodium citrate buffer pH6, primary antibodies were labeled by using EnVision, HRP-labeled System (cod. K4001, Dako, CA, USA) and visualized by 3’-3’diaminobenzidine substrate (DAB). The nuclei were highlighted by counterstaining with Mayer’s hematoxylin. Collagen deposition was evidenced by Picro-Sirius Red staining as previously described (24). In some experiments, hematoxylin/eosin staining was performed on murine liver sections obtained by mice fed with CDAA diets: these sections were scored blind for steatosis and lobular inflammation.

#### Biochemical Analyses

Plasma alanine aminotransferase (ALT) was determined by spectrometric kits supplied by Gesan Produzione SRL (Campobello di Mazara, Italy).

#### Quantitative Real Time PCR (qRT-PCR)

RNA extraction, complementary DNA synthesis, quantitative real-time PCR reactions were performed as previously described (31). RNA was extracted from mouse livers and from human cells with TRI reagent and retro-transcribed using the iScript™ cDNA Synthesis Kit (BioRad, Berkeley, CA, USA) according to the manufacturer’s instruction. Realtime PCR was performed using either Miniopticon ThermoCycler Instrument (Biorad, Berkeley, CA, USA) or, for some genes (murine CD9, TREM-2 and Gal-3), a Techne TC-312 termacycler (Techne) using TaqMan Gene Expression Master Mix and TaqMan Gene Expression probes (Applied Biosystem Italia). Oligonucleotide sequence of primers used for RT-PCR were listed in **Supplementary Material**.

#### Western Blotting

Western blotting analysis was performed as previously described (24, 31). Briefly, total cell lysates were subjected to sodium dodecyl sulfate-polyacrylamide gel-electrophoresis on 10% acrylamide gels, incubated with desired primary antibodies, then with peroxidase-conjugated anti-mouse or anti-rabbit immunoglobulins in Tris-buffered Saline-Tween containing 2% (w/v) non-fat dry milk and finally developed with the ECL reagents according to manufacturer’s instructions. Sample loading was evaluated by re-blotting the same membrane with the un-phosphorylated form of protein or with β-actin antibody (24, 31).

#### Detection of Intracellular Generation of ROS

Combination of DCFH-DA (2′,7′-Dichlorofluorescin diacetate) technique and flow cytometric analysis: THP-1 cells differentiated to macrophages were seeded in P35 dishes (5 × 10^5^ cells/dish), cultured for 24 hrs and exposed to hrSB3 200ng/ml for 30 min, 1hr and 3hrs before addition of 5 μM DCFH-DA (15 min of incubation in the dark). Cells were rapidly washed with PBS, collected by trypsinization, briefly centrifuged (1600 rpm for 5 min) and re-suspended in PBS for analysis. Detection of DCF green fluorescence (FL1) was performed on at least 5000 cells per sample with a FACScan equipped with a 488 nm argon laser using the CellQuest software (version 1.0.264.21, Becton-Dickinson, Milano, Italy). The peak of FL1 intensity of DCFH-DA-stained control cells grown without SB3 was set to channel 101 and retained for all measurements (31).

#### ELISA Quantification

Specific ELISA kits were used to quantify TGFβ1, VEGF, TNFα and IL-1β released in the culture medium by THP-1 cells differentiated to macrophages, exposed or not to hrSB3 for different times, following the instructions of the supplier company (Invitrogen, Thermofisher Scientific Italia, Monza MI, Italy).

#### Statistical Analysis

Statistical analyses were performed by SPSS statistical software (SPSS Inc. Chicago IL, USA) using one-way ANOVA test with Turkey’s correction for multiple comparisons or Kruskal-Wallis test for non-parametric values. Significance was taken at the 5% level. Normality distribution was assessed by the Kolmogorov-Smirnov algorithm. Data from *in vitro* experiments represent means ± SEM obtained from at least three independent experiments. Luminograms and morphological images are representative of at least three experiments with similar results.

## Results

### Hepatocyte-Specific Manipulation of SB3 Expression Affects Inflammatory Response in Two Distinct Protocols of Murine NASH

**Figure 1:**
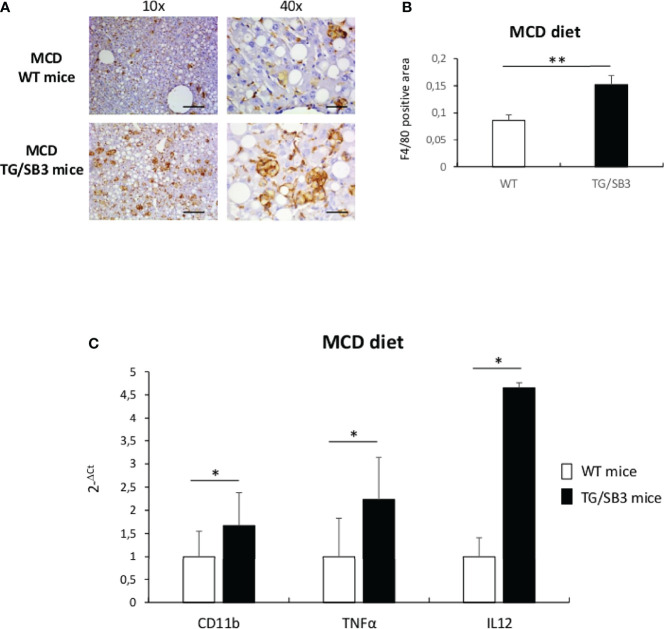
**(A–C) (A)** Immunoistochemistry analysis for F4/80 on liver specimens obtained from C57Bl6J WT mice and C57Bl6J transgenic mice for SB3 (TG/SB3) fed on MCD diet for 8 weeks. Magnification 10x, scale bar 200µm, magnification 40x, scale bar 50µm. **(B)** ImageJ software analysis performed to evaluate the amount of F4/80 positive area in C57Bl6J WT mice and C57Bl6J transgenic mice for SB3 (TG/SB3) fed on MCD diet for 8 weeks. **p < 0.01 versus WT mice. **(C)** Quantitative real time PCR analysis of CD11b, TNFα and IL12 in C57Bl6J WT mice and transgenic mice for SB3 (TG/SB3) fed on MCD diet for 8 weeks. *p < 0.05 versus WT mice.

Previous studies have evidenced that HIF2α-dependent signals are important in modulating inflammatory responses associated with the progression of NAFLD/NASH (24). Since SB3 is strictly associated with HIF2α regulation (25), here we investigated whether, beside a pro-fibrogenic action, SB3 might have pro-inflammatory role in NAFLD/NASH. In preliminary experiments, we first analyzed liver samples from either WT or TG/SB3 mice fed for 8 weeks on the MCD diet for inflammatory infiltration. Immunohistochemistry analysis for the murine macrophage marker F4/80 revealed that macrophage infiltration was greatly increased in the liver of TG/SB3 mice as compared to WT mice fed on MCD diet (**Figure 1A**). According to previous observations (23, 37), the macrophages accumulating in the livers of MCD-fed mice had a foamy appearance and formed aggregates with a crown-like structures (**Figure 1A**). Histo-morphometric analysis confirmed that the prevalence of these aggregates was significantly increased in TG/SB3 (**Figure 1B**). Accordingly, transcripts for markers of pro-inflammatory macrophage activation such as CD11b, TNFα, and IL-12 were significantly higher in the liver of TG/SB3 mice than in those of WT mice fed on MCD diet (**Figure 1C**).

**Figure 2:**
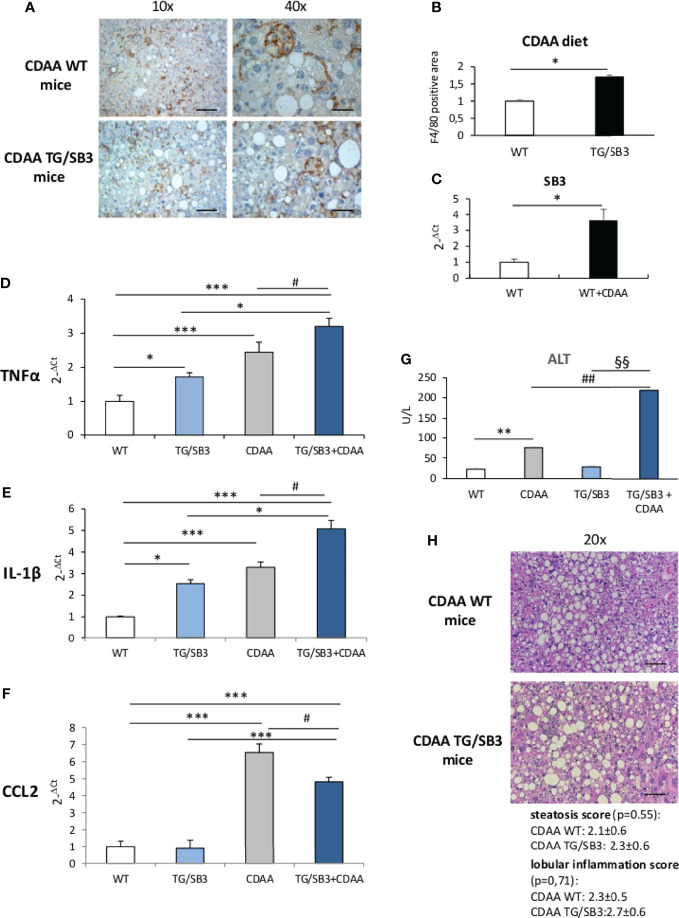
**(A–H) (A)** Immunohistochemistry analysis for F4/80 on liver specimens obtained from C57Bl6J WT mice and transgenic mice for SB3 (TG/SB3) fed on CDAA for 12 weeks. Magnification 10x, scale bar 200µm, magnification 40x, scale bar 50µm. **(B)** ImageJ software analysis performed to evaluate the amount of F4/80 positive area in C57Bl6J WT mice and transgenic mice for SB3 (TG/SB3) fed on CDAA for 12 weeks. *p < 0.05 versus WT mice. **(C)** Quantitative real time PCR analysis of SB3 in C57Bl6J WT mice fed on control (WT) or CDAA diet (WT+CDAA). *p < 0.05 versus WT mice. Quantitative real time PCR analysis of TNFα **(D)**, IL-1β **(E)** and CCL2 **(F)** in C57Bl6J WT mice and transgenic mice for SB3 (TG/SB3) fed on control CSAA diet (WT and TG/SB3) or CDAA diet for 12 weeks (CDAA and TG/SB3+CDAA). ***p < 0.001 and *p < 0.05 versus WT mice fed on control CSAA diet (WT) or versus TG/SB3 mice fed on CSAA diet; ^#^p < 0.05 versus C57Bl6J WT mice fed on CDAA diet (CDAA). **(G)** Serum levels of alanine amino transferase (ALT) analyzed as a parameter of parenchymal injury in C57Bl6J WT mice and transgenic mice for SB3 (TG/SB3) fed on control CSAA diet (WT and TG/SB3) or CDAA diet for 12 weeks (CDAA and TG/SB3+CDAA). **p < 0.01 versus WT mice fed on control CSAA diet (WT), ^##^p < 0.01 versus WT mice fed on CDAA diet, ^§§^p < 0.01 versus TG/SB3 mice fed on CSAA diet. **(H)** Hematoxylin eosin staining and score of steatosis and lobular inflammation in WT e TG/SB3 mice fed on CDAA diet for 12 weeks to evaluate steatosis and inflammation. Magnification 20x, scale bar 100µm.

To further evaluate the effects of SB3 in NASH-associated inflammation, additional experiments were performed by inducing steatohepatitis with choline-deficient aminoacid-refined (CDAA) diet that, differently from the MCD diet, does not cause malnutrition and more closely resembles human NASH (24). We observed that in wild type mice receiving the CDAA diet for 12 weeks the development of steatohepatitis and the increased presence of macrophage crown-like aggregated (**Figures 2A, B**) associated with a three folds up-regulation in the liver transcripts for SB3 (**Figure 2C**). The administration of the same diet to TG/SB3 mice led to a further increase in the prevalence of liver crown-like structures (**Figure 2A**) and a significant up-regulation of liver transcripts for TNFα and IL-1β (**Figures 2D, E**) as compared to CDAA-fed WT mice, while the expression of the monocyte chemokine CCL2 was unchanged (**Figure 2F**). In line with these findings, the release of transaminase was higher in TG/SB3 mice than WT littermates (**Figure 2G**). No significant differences were instead appreciable in the histopathological scores for steatosis (2.1 ± 0.6 vs 2.3 ± 0.6; p=0.55) and lobular inflammation (2.3 ± 0.5 vs 2.7 ± 0.6; p=0.71) (**Figure 2H**). It should be noted that TNFα and IL-1β hepatic transcript levels, but not those of CCL2, were already up-regulated in the liver of TG/SB3 mice vs WT mice fed on control CSAA diet, without signs of hepatic injury (**Figures 2D–F**).

**Figure 3:**
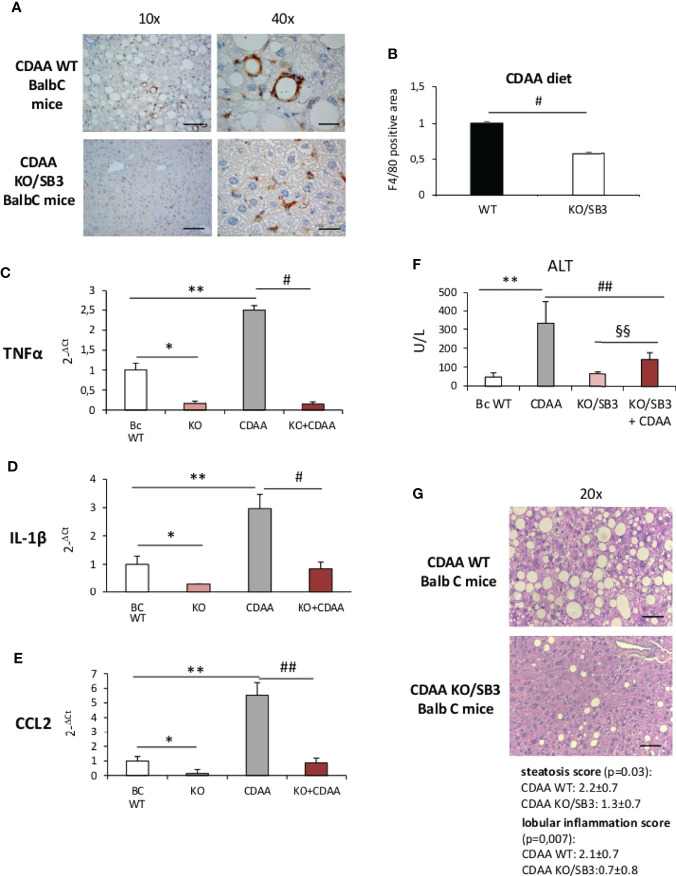
**(A–G) (A)** Immunohistochemistry analysis for F4/80 on liver specimens obtained from BalbC WT mice and knock out mice for SB3 (KO/SB3) fed on CDAA diet for 12 weeks. Magnification 10x, scale bar 200µm, magnification 40x, scale bar 50µm. **(B)** ImageJ software analysis performed to evaluate the amount of F4/80 positive area. ^#^p < 0.05 versus BalbC control mice. Quantitative real time PCR analysis of TNFα **(C)**, IL-1β **(D)** and CCL2 **(E)** in WT BalbC mice and knock out mice for SB3 (KO/SB3) fed on control CSAA diet (BC WT and KO) or CDAA diet for 12 weeks (CDAA and KO+CDAA). *p < 0.05, **p < 0.01 versus BalbC WT mice fed on control CSAA diet (BC WT); ^#^p < 0.05 ^##^p < 0.01 versus BalbC WT mice fed on CDAA (CDAA). **(F)** Serum levels of alanine amino transferase (ALT) analyzed as a parameter of parenchymal injury in BalbC WT mice and knock out mice for SB3 (KO/SB3) fed on control CSAA diet (BC WT and KO/SB3) or CDAA diet for 12 weeks (CDAA and KO/SB3+CDAA). **p < 0.01 versus WT mice fed on control CSAA diet (BC WT), ^##^p < 0.01 versus BalbC WT mice fed on CDAA diet (CDAA), ^§§^ p < 0.01 versus KO/SB3 mice fed on CSAA diet. **(G)** Hematoxylin eosin staining and score of steatosis and lobular inflammation in WT e KO/SB3 mice fed on CDAA diet for 12 weeks to evaluate steatosis and inflammation. Magnification 20x, scale bar 100µm.

Further support to the involvement of SB3 in promoting macrophage-mediated inflammatory responses was offered by experiments performed on mice deficient of the reactive site loop of SerpinB3a gene (KO/SB3). In these animals, we observed that, following 12 weeks on the CDAA diet, the lack of SB3 anti-protease activity greatly lowered the formation of macrophage crown-like aggregates (**Figures 3A, B**) and dramatically reduced hepatic transcripts of TNFα, IL-1β and CCL2 (**Figures 3C–E**). Consistently, the severity of steatohepatitis as evaluated by transaminase release (**Figure 3F**) and by histopathological scores for steatosis (2.2 ± 0.7 vs 1.3 ± 0.7; p=0.03) and lobular inflammation (2.1 ± 0.7 vs 0.7 ± 0.8; p=0.007) was significantly improved in the absence of SB3 (**Figure 3G**). Interestingly, also the extension of liver fibrosis as evaluated by collagen staining with Sirius Red was appreciably reduced in KO/SB3 mice (**Supplementary Figure 1A**). A consistent decrease in steatosis, F4/80 positive cells and Sirius red staining was also evident in KO/SB3 mice receiving the MCD diet (**Supplementary Figures 1B–D**).

### SB3 Can Directly Activate Macrophage Responses

**Figure 4:**
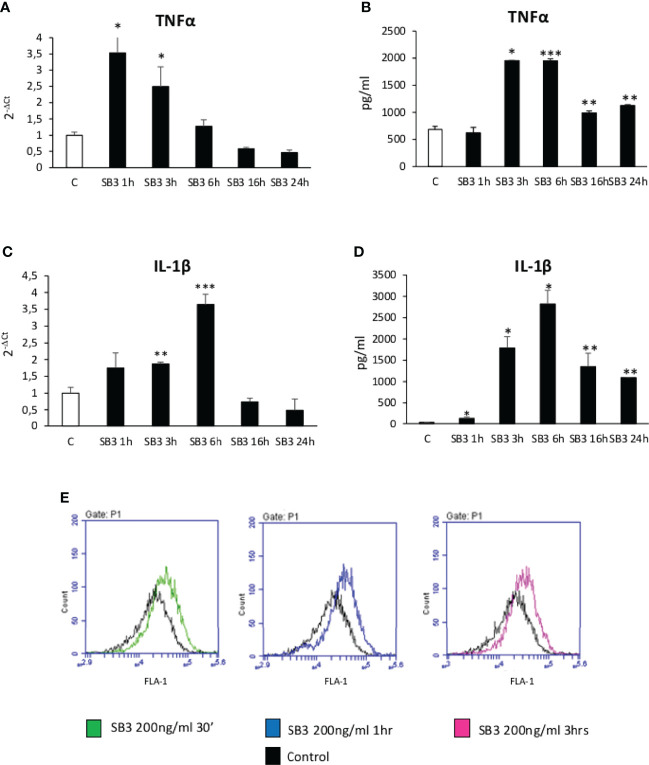
**(A–E)** Time course analysis of transcript levels by quantitative real time PCR **(A, C)** as well as of protein levels by ELISA **(B, D)** of TNFα and IL-1β in human differentiated THP1 cells exposed or not to hrSB3 200ng/ml (SB3) up to 24 hours. *p < 0.05, **p < 0.01, ***p < 0.001 versus control cells. **(E)** DCFH-DA fluorescence in flow cytometry analysis to detect intracellular generation of reactive oxygen species induced by exposure of cells to hrSB3 200ng/ml for the indicated time points.

**Figure 5:**
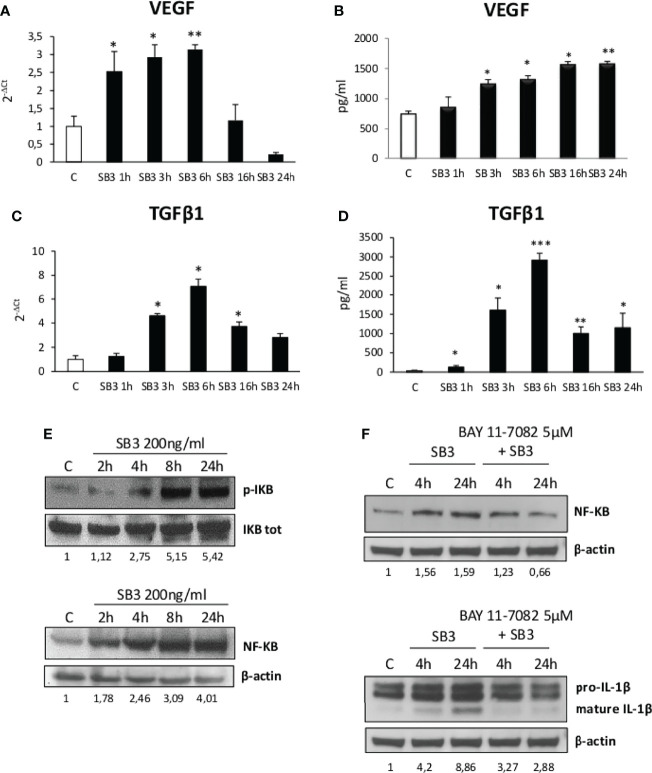
**(A–F)** Time course analysis of transcript levels by quantitative real time PCR **(A, C)** as well as of protein levels by ELISA **(B, D)** of VEGF and TGFβ1 in human differentiated THP1 cells exposed or not to hrSB3 200ng/ml (SB3) up to 24 hours. *p < 0.05, **p < 0.01, ***p < 0.001 versus control cells. **(E)** Western blotting analysis of phosphorylated IKB and of protein levels of NF-kB in human differentiated THP-1 cells exposed or not to hrSB3 200ng/ml (starting from 2 hrs up to 24 hrs). Equal loading was confirmed by re-probing the same membrane with the un phosphorylated protein IKB or with β-actin. Values obtained from band densitometry analysis, using ImageJ software, are reported. **(F)** Western blot analysis of protein levels of NFkB and IL-1β in human THP-1 cells exposed to hrSB3 200ng/ml for 4 and 24 hrs or pre-treated with the inhibitor of IKK protein, BAY 11-7082 5µM and then exposed to hrSB3 200ng/ml for 4hrs and 24 hrs. Equal loading was confirmed by re-probing the same membrane with β-actin. Values obtained from band densitometry analysis, using ImageJ software, are reported.

From the above results we investigated by which mechanism SB3 may influence macrophage functions. In preliminary experiments we first tested *in vitro* the response of either human monocytes obtained from peripheral blood or undifferentiated human THP-1 cells to the addition of hrSB3 observing that, in both cell types, hrSB3 strongly up-regulated the expression of TNFα, IL-1β and IL-12 (**Supplementary Figures 2A–F**). We next tested the ability of hrSB3 to influence the response of human THP-1 cells differentiated into macrophages by 48 hours of incubation with 50 nM phorbol-12-myristate-13-acetate (PMA). Here again, differentiated THP-1 cells receiving hrSB3 underwent an appreciable and sustained up-regulation of TNFα and IL-1β transcripts (**Figures 4A, C**) and proteins (**Figures 4B, D**) along with an increase in the intracellular generation of ROS (**Figure 4E**). Interestingly, we found that hrSB3 stimulation of THP-1-derived macrophages led also to a significant increase at both mRNA (**Figures 5A, C**) and protein (**Figures 5B, D**) levels of pro-fibrogenic mediators VEGF-A and TGFβ1, indicating that these cells were acquiring the mixed pro-inflammatory and pro-fibrogenic phenotype characteristic of NASH-associated MoMFs (15–17). Since at present the receptor for SB3 on target cells has not yet been characterized, we next investigated whether hrSB3 may operate as pro-inflammatory mediator by up-regulating the NF-kB transcription factor. Results from these experiments showed that hrSB3 led to a rapid and progressive stimulation of NF-kB as well as of p-IKB, the NF-kB inhibitor that, when phosphorylated, is dissociated from the nuclear factor favoring NF-kB activation and then its nuclear translocation (**Figure 5E**); the NF-kB stimulation was blocked by cell pre-treatment with the IKK pharmacological inhibitor BAY 11-7082. The addition of the IKK inhibitor also reduced IL-1β production in the same cells (**Figure 5F**).

### SB3 Influences NAM Markers in Mice With NASH

**Figure 6:**
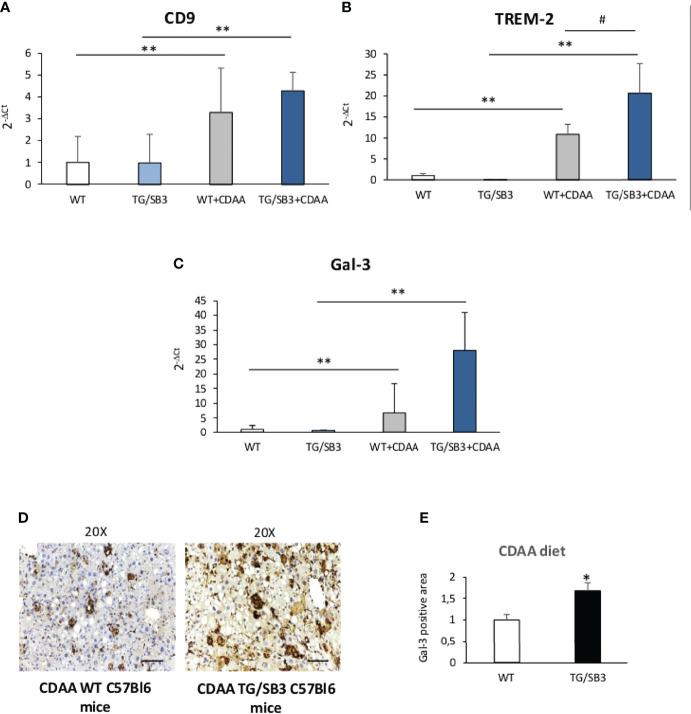
**(A–E)** Quantitative real time PCR analysis of CD9, **(A)** TREM-2 **(B)** and Gal-3 **(C)** in control C57Bl6J mice and transgenic mice for SB3 fed on control diet (WT and TG/SB3) or CDAA diet for 12 weeks (WT+CDAA and TG/SB3+CDAA). **p < 0.01 versus C57Bl6J control mice fed with control diet (WT) ^#^p < 0.05 versus C57Bl6J control mice fed with CDAA (WT+CDAA). **(D)** Immunohistochemistry analysis for Gal-3 on liver specimens obtained from C57Bl6J WT mice and transgenic mice for SB3 (TG/SB3) fed on CDAA for 12 weeks. Magnification 20x, scale bar 100µm. **(E)** ImageJ software analysis was performed to evaluate the amount of Gal-3 positive area in C57Bl6J WT mice and transgenic mice for SB3 (TG/SB3) fed on CDAA for 12 weeks. *p < 0.05 versus WT mice.

**Figure 7:**
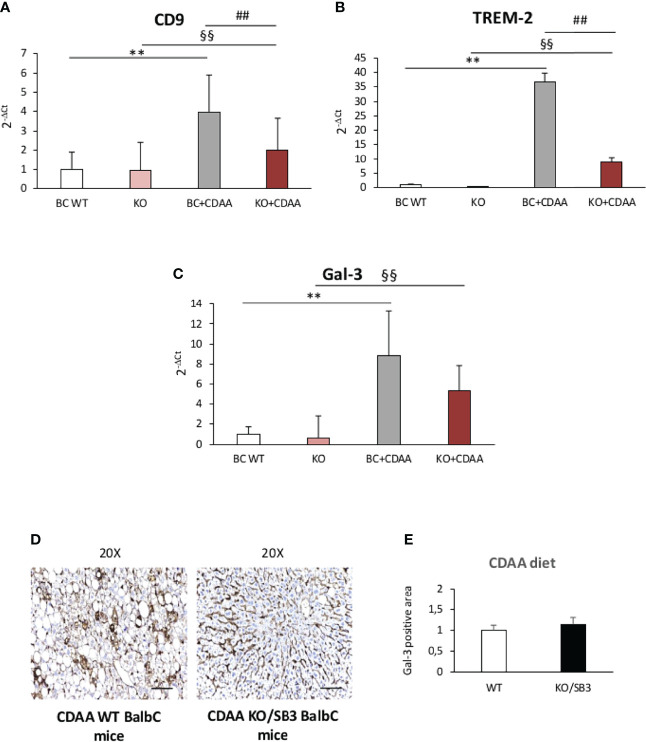
**(A–E)** Quantitative real time PCR analysis of CD9, **(A)** TREM-2 **(B)** and Gal-3 **(C)** in control BalbC mice and knock out mice for SB3 fed on control diet (BC WT and KO) or CDAA diet for 12 weeks (BC+CDAA and KO+CDAA). **p < 0.01 versus BalbC control mice fed with control diet (BC WT); ^§§^p < 0.01 versus BalbC control mice fed with control diet (KO), ^##^p < 0.01 versus BalbC control mice fed with CDAA diet (BC+CDAA). **(D)** Immunohistochemistry analysis for Gal-3 in liver specimens obtained from WT BalbC mice and KO/SB3 BalbC mice fed on CDAA diet for 12 weeks. Magnification 20x, scale bar 100µm. **(E)** ImageJ software analysis was performed to evaluate the amount of Gal-3 positive area in BalbC WT mice and knock out mice for SB3 (KO/SB3) fed on CDAA for 12 weeks.

From these observations and the notion that NAMs are involved in forming crown-like structures in NASH livers (21), we went to investigate whether SB3 might influence the expression of NAM markers such as TREM2 and CD9 along with that of the fibrogenic Galectin-3 (Gal-3) (38–41) which is specifically associated with the macrophage phenotype (18–21). **Figure 6** shows that all these three markers were up-regulated in WT mice fed on CDAA diet and were further significantly enhanced in mice overexpressing SB3 in hepatocytes (**Figures 6A–C**). Furthermore, immunohistochemistry demonstrated that the increased expression of Gal-3 mainly involved macrophages aggregates (**Figures 6D, E**). Conversely, CD9 and TREM2 transcripts were significantly down-regulated in the livers of KO/SB3 mice fed on CDAA diet as compared to the respective WT controls (**Figures 7A, B**). In the same animals the decrease Gal-3 mRNA did not reach statistical significance (**Figure 7C**), however immunohistochemistry demonstrated the disappearance of Gal-3 positive macrophage aggregates in KO/SB3 mice receiving the CDAA diet (**Figures 7D, E**). Altogether these data indicate that SB3 contributes to sustain macrophage pro-inflammatory responses during the progression of NASH.

## Discussion

It is now well established that hepatic macrophages play a key role in the progression of NAFLD by contributing to sustain both lobular inflammation and hepatic stellate cells (HSCs) activation to matrix producing myofibroblasts (15–17). In this setting great attention has been payed to the mechanisms that promote Kupffer cells activation at the onset of steatohepatitis as well as to the factors involved in favoring macrophage recruitment within the liver (15–17). What is less understood is the network of signals that contribute to maintain the pro-inflammatory and pro-fibrogenic activities of hepatic macrophages during the disease evolution. This is of particular interest on the light of recent findings showing that monocyte-derived macrophages recruited in NASH livers undergo to a specific phenotype reprogramming with increased expression of TREM2 and CD9 (19–21). Interestingly, the prevalence of these TREM2-positive macrophages also known as NAM is strongly associated with the severity of steatosis, inflammation, hepatocyte ballooning, and fibrosis (20).

Here we show that the protease inhibitor SB3 plays an important role among the signals that contributes to promote the pro-inflammatory phenotype of liver macrophage in NASH. The production of SB3 is strongly up-regulated in steatotic and hypoxic hepatocytes in relation to signaling mediated by HIF2α (23, 25, 28–31). In a previous study we provided experimental evidence of the pro-fibrogenic action of SB3 by showing that mice overexpressing SB3 in hepatocytes and exposed to different protocols of chronic liver injury were characterized by an enhanced hepatic collagen deposition in relation to SB3 capability to directly up-regulate the transcription of fibrogenic genes in human activated, myofibroblast-like, hepatic stellate cells (HSC/MFs) or human stellate cell line (LX2 cells) (31). The evidence supporting the role of SB3 as a pro-inflammatory mediator comes from experiments in which mice genetically manipulated to carry hepatocyte specific SB3 overexpression (TG/SB3) or deficient in SB3 (KO/SB3) were fed with two different NASH-inducing dietary protocols. In particular, we have observed that SB3 overexpression not only leads to increased fibrosis, as previously reported (31), but also to an increase in transcript levels for TNFα and IL-1β and to recruitment of infiltrating monocyte-derived macrophages that accumulate mainly in crown-like aggregates, a feature which is characteristic of human advanced NAFLD (42, 43). Conversely, SB3 deletion greatly reduces lobular inflammation markers, macrophage aggregates and the severity of steatohepatitis.

Although the receptor for SB3 has not yet been identified, *in vitro* experiments support these observations by showing that hrSB3 can directly activate human monocytes from peripheral blood as well as undifferentiated or PMA-differentiated human THP1 macrophages. In these cells, SB3-mediated activation results in a NF-kB-dependent up-regulation of pro-inflammatory cytokines and ROS production. Such a pro-inflammatory action of SB3 on macrophages shows several analogies with that of the HRG which is similarly produced by hepatocytes in response to HIF2α-mediated signals (23, 26). Interestingly, SB3 addition to differentiated THP1 macrophages also induces a significant up-regulation of VEGF and TGFβ1, two cytokines that are related to macrophage pro-fibrogenic activity. This is consistent with previous observations concerning the role of SB3 in stimulating the progression of liver fibrosis (31). Thus, the data from the present study suggest that SB3 can operate in NASH as a peculiar hepatocyte-released mediator being able to induce a macrophage mixed pro-inflammatory/pro-fibrogenic phenotype that characterizes the evolution of CLD, including NAFLD/NASH (15–17, 44, 45). The latter phenotype has been reported to sustain the fibrogenic progression of chronic diseases by releasing several mediators, including TGFβ1, FGF, TGFα, Activin 1, PDGF, IGF-1, VEGF-A and Galectin 3 (45).

The action of SB3 in NASH is further emphasized by the fact that hepatocyte SB3 production influences the hepatic levels of NAM markers TREM2, CD9 and Galectin 3. These markers have been reported to identify a peculiar subset of hepatic macrophages emerging in conditions of either human of murine progressive NAFLD (19–21, 46, 47) showing similarities with TREM2 CD9 expressing scar-associated macrophages (SAMs). This suggests the possibility that, during NAFLD progression, SB3 might also contribute to the emergence of NAMs and/or SAMs. Such a SB3 action on liver macrophages is not in contrast with previous observations concerning the pro-fibrogenic role of SB3 (31) since the macrophage phenotype induced by SB3 involves the release pro-fibrogenic mediators such as Gal-3, VEGF and TGFβ1 that can sustain liver matrix production by HSC/MFs.

Although more research is needed to confirm this hypothesis, the data provided by the present study indicate that SB3 can be added to the list of peptide mediators contributing to NASH pathogenesis. Some of these mediators have been collectively termed “hepatokines”, being defined as proteins synthetized and secreted by hepatocytes that can influence metabolic processes through autocrine, paracrine and endocrine signaling, playing a central role in orchestrating whole-body energy metabolism (48–50). In particular, the development of fatty liver has been reported to promote the secretion of hepatokine such hepassocin, fetuins A and B and fibroblast growth factor 21 (FGF21) (49), that are also involved in causing liver inflammation (49, 50). Although SB3 has not been so far included in the list of hepatokines, the evidence emerging on their contribution to NAFLD progression suggest the possibility to consider SB3, which is selectively expressed by hepatocytes under CLD conditions, as a putative novel hepatokine.

## Conclusions

Data reported in the present study provide novel evidence that SB3, produced and released by activated/injured hepatocytes, can operate as a pro-inflammatory mediator in NASH efficiently contributing to disease progression.

## Data Availability Statement

The original contributions presented in the study are included in the article/**Supplementary Material**. Further inquiries can be directed to the corresponding authors.

## Ethics Statement

Human blood samples employed to purify monocytes were obtained under informed consent and the study protocol, which conformed to the ethical guidelines of the 1975 Declaration of Helsinki, was approved by the Ethics Committee of the Azienda Ospedaliera-Università, Padova, Italy. The patients/participants provided their written informed consent to participate in this study. The animal study was reviewed and approved by Animal Ethical Committee of University of Padua and by the Animal Investigation Committee of the Italian Ministry of Health.

## Author Contributions

Conceptualization EN, AC, PP, EA, and MP; methodology, EN, AC, GV, MG, SQ, SC, CB, CT, MM, FP, SS, AP, MR, AB, and BF; software, EN, AC, SS, and EA; validation, EA, PP, and MP; formal analysis EN, AC, PP, EA, and MP; investigation, EN, AC, GV, CT, CB, MM, and FP; resources, EA, PP, and MP; data curation, EN, AC, GV, PP, and MP; writing—original draft preparation, PP and MP; writing—review and editing, EN, EA, and PP; visualization, EN, AC, PP, and MP; supervision, EA, PP, and MP; project administration, PP and MP; funding acquisition, EN, PP, and MP. All authors have read and agreed to the published version of the manuscript.

## Funding

The research leading to these results has received funding from: (a) Associazione Italiana per la Ricerca sul Cancro (AIRC) under IG2017-ID 20361 – P.I. Maurizio Parola (MP); (b) the University of Torino (Fondo di Ateneo ex 60% - EN, MP); (c) University of Padova project No CPDA110795-P.P.

## Conflict of Interest

The authors declare that the research was conducted in the absence of any commercial or financial relationships that could be construed as a potential conflict of interest.

## Publisher’s Note

All claims expressed in this article are solely those of the authors and do not necessarily represent those of their affiliated organizations, or those of the publisher, the editors and the reviewers. Any product that may be evaluated in this article, or claim that may be made by its manufacturer, is not guaranteed or endorsed by the publisher.

## Abbreviations

NAFLD, non-alcoholic fatty liver disease; NASH, non-alcoholic steatohepatitis; SB3, SerpinB3; hrSB3, human recombinant SB3; HRG, histidine rich glycoprotein; MCD, methionine/choline deficient; CDAA, choline-deficient, L-amino acid defined; TREM2, triggering receptor expressed on myeloid cells-2; HCC, hepatocellular carcinoma; MoMFs, monocyte-derived macrophages; HSCs, hepatic stellate cells; MFs, hepatic myofibroblasts; NAMs, NASH-associated macrophages; LAMs, lipid-associated macrophages; ROS, reactive oxygen species; HRG, histidine-rich glycoprotein; CLD, chronic liver diseases; HSC/MFs, myofibroblast-like, hepatic stellate cells; MCS, methionine choline supplemented diet; CSAA, choline-supplemented and aminoacid-refined diet; ECL, Enhanced chemiluminescence; PBS, phosphate saline buffer; NaCl, sodium chloride; BSA, bovine serum albumin; NaN3, sodium azide; PMA, phorbol 12-myristate 13-acetate; DAB, 3’-3’diaminobenzidine substrate; ALT, alanine aminotransferase; qRT-PCR, quantitative Real Time PCR; DCFH-DA, 2′,7′-Dichlorofluorescin diacetate; SAMs, scar-associated macrophages; FGF21, fibroblast growth factor 21.
