# Supplementary figures and images for: SerpinB3 as a Pro-Inflammatory Mediator in the Progression of Experimental Non-Alcoholic Fatty Liver Disease
Source: Front Immunol. 2022 Jul 8;13:910526. doi: 10.3389/fimmu.2022.910526 (PMC9304805; doi:10.3389/fimmu.2022.910526)

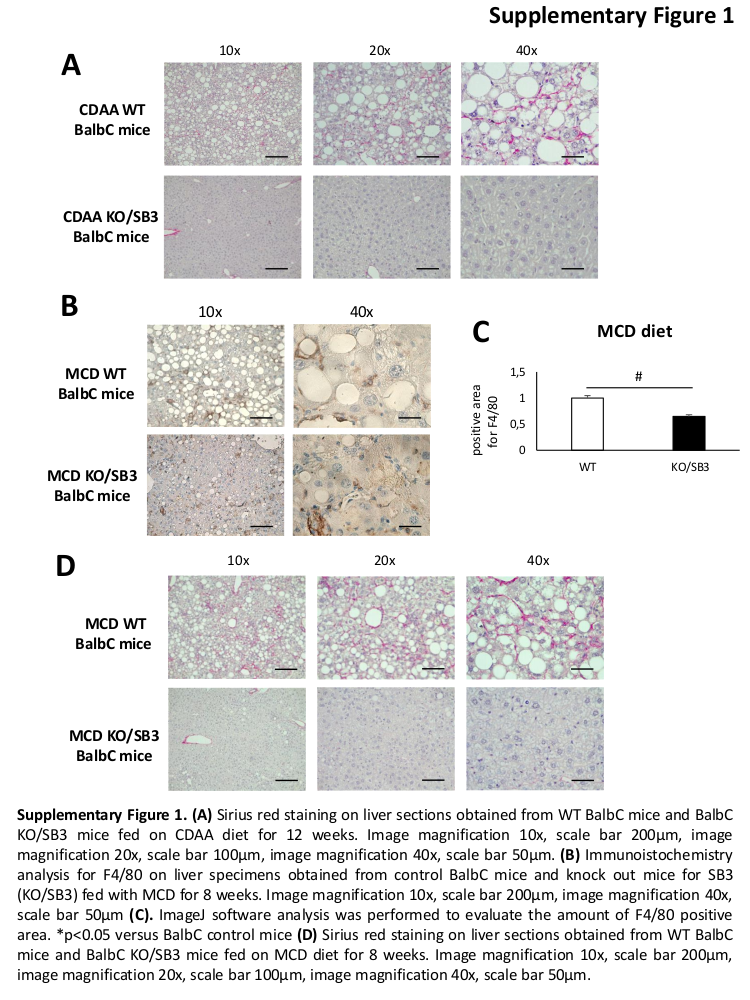

Supplement: Supplementary file 1 [file Image_1.tif]

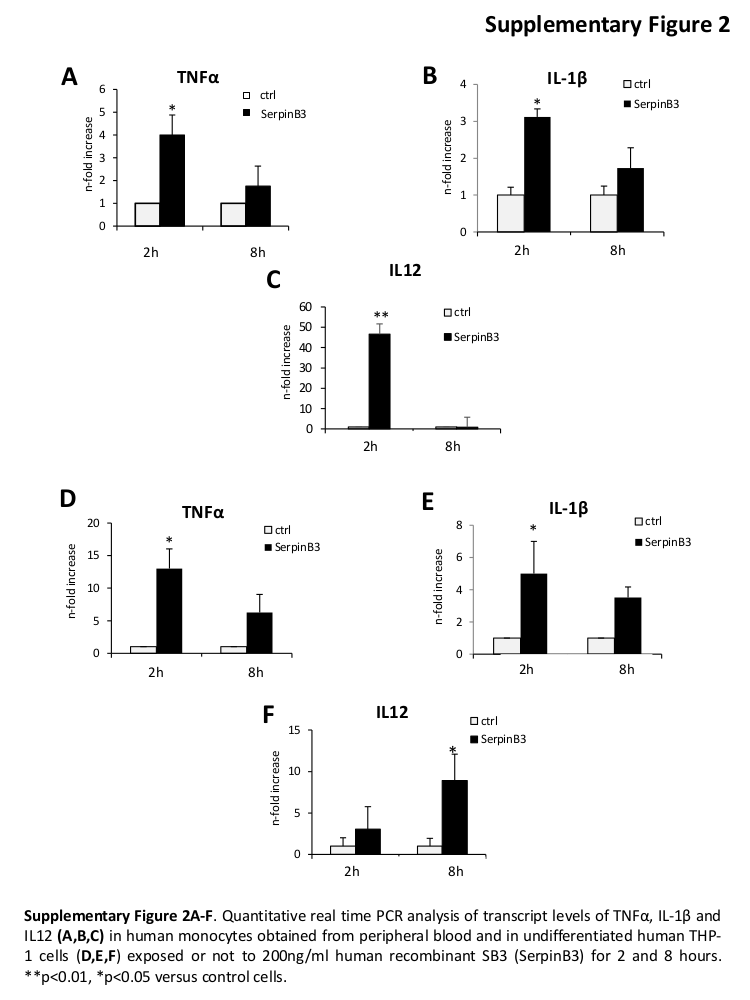

Supplement: Supplementary file 2 [file Image_2.tif]
