# Supplementary material for: SerpinB3 as a Pro-Inflammatory Mediator in the Progression of Experimental Non-Alcoholic Fatty Liver Disease
Source: Front Immunol. 2022 Jul 8;13:910526. doi: 10.3389/fimmu.2022.910526 (PMC9304805; doi:10.3389/fimmu.2022.910526)
Supplement: Supplementary file 3 [file Table_1.docx]

**Supplementary material:**

| **Gene** | **Primers** |
| --- | --- |
| *TBP* (murine gene) | (FW) 5’CACATCACAGCTCCCCACCA 3’  (RV) 5’AGCGGAGAAGATGCTGGAAAC 3’ |
| *IL-1β* (murine gene) | (FW) 5’GAAATGCCACCTTTTGACA 3’  (RV) 5’TTGGAAGCAGCCCTTCATCTT 3’ |
| *TNF-α* (murine gene) | (FW) 5’ACGGCATGGATCTCAAAGAC 3’  (RV) 5’GTGGGTGAGGAGCACGTAGT 3’ |
| *CCL2* (murine gene) | (FW) 5’CCCAATGAGTAGGCTGGAGA 3’  (RV) 5’TCTGGACCCATTCCTTCCTTG 3’ |
| *CD11b* (murine gene) | (FW) 5’TACCGGAAGGAATTCAGCAAG 3’  (RV) 5’TAGCAGGAAAGATGGGATGG 3’ |
| *IL12* (murine gene) | (FW) 5’AGGAACCTGAAACTCCCCAG 3’  (RV) 5’GTCAAATCCAGAACATGCCGC 3’ |
| *SB3* (human gene) | (FW) 5’ GCAAATGCTCCAGAAGAAAG 3’  (RV) 5’ CGAGGCAAAATGAAAAGATG 3’ |
| *CCL2* (human gene) | (FW) 5’CCCCAGTCACCTGCTGTTAT 3’  (RV) 5’AGATCTCCTTGGCCACAATG 3’ |
| *IL-1β* (human gene) | (FW) 5’ TGAAAGCTCTCCACCTCCAG 3’  (RV) 5’ CACGCAGGACAGGTACAGAT 3’ |
| *TNF-α* (human gene) | (FW) 5’ AACCTCCTCTCTGCCATCAA 3’  (RV) 5’ GGAAGACCCCTCCCAGATAG 3’ |
| *VEGF* (human gene) | (FW) 5’ CCCACTGAGGAGTCCAACAT 3’  (RV) 5’ TTTCTTGCGCTTTCGTTTTT 3’ |
| *GAPDH* (human gene) | (FW) 5’ TGGTATCGTGGAGGACTCATGGAC3’  (RV) 5’ ATGCCAGTGAGCTTCCCGTTCAGC 3’ |

**Table 1.** List and sequences of murine and human primers used for quantitative real time PCR.
